# Cross-modal attention model integrating tongue images and descriptions: a novel intelligent TCM approach for pathological organ diagnosis

**DOI:** 10.3389/fphys.2025.1580985

**Published:** 2025-04-23

**Authors:** Quan Gan, Chen Wang, Zhaoman Zhong, Jiaying Wu, Qiwei Ge, Lei Shi, Jiaqing Shang, Chuanxia Liu

**Affiliations:** ^1^ School of Computer Engineering, Jiangsu Ocean University, Lianyungang, China; ^2^ The Graduate school of East Asian Studies, Yamaguchi University, Yamaguchi-shi, Japan; ^3^ School of Foreign Languages, Jiangsu Ocean University, Lianyungang, China

**Keywords:** tongue diagnosis, pathological organ, tongue images analysis, textual descriptions, cross-modal attention

## Abstract

**Introduction:**

Tongue diagnosis is a fundamental technique in traditional Chinese medicine (TCM), where clinicians evaluate the tongue’s appearance to infer the condition of pathological organs. However, most existing research on intelligent tongue diagnosis primarily focuses on analyzing tongue images, often neglecting the important descriptive text that accompanies these images. This text is an essential component of clinical diagnosis. To overcome this gap, we propose a novel Cross-Modal Pathological Organ Diagnosis Model that integrates tongue images and textual descriptions for more accurate pathological classification

**Methods:**

Our model extracts features from both the tongue images and the corresponding textual descriptions. These features are then fused using a cross-modal attention mechanism to enhance the classification of pathological organs. The cross-modal attention mechanism enables the model to effectively combine visual and textual information, addressing the limitations of using either modality alone

**Results:**

We conducted experiments using a self-constructed dataset to evaluate our model’s performance. The results demonstrate that our model outperforms common models regarding overall accuracy. Additionally, ablation studies, where either tongue images or textual descriptions were used alone, confirmed the significant benefit of multimodal fusion in improving diagnostic accuracy.

**Discussion:**

This study introduces a new perspective on intelligent tongue diagnosis in TCM by incorporating visual and textual data. The experimental findings highlight the importance of cross-modal feature fusion for improving the accuracy of pathological diagnosis. Our approach not only contributes to the development of more effective diagnostic systems but also paves the way for future advancements in the automation of TCM diagnosis.

## 1 Introduction

Traditional Chinese Medicine (TCM) is a longstanding medical system that has gained global recognition as a complementary and alternative therapy (Gan et al., 2021a; Gan et al., 2021b). Among TCM’s diagnostic methods, tongue diagnosis stands out as a unique and essential approach, having been extensively validated in clinical practice for its ability to reveal various pathological conditions (Zhang et al., 2022; Liu and Han, 2013). With the rapid development of medical technology and artificial intelligence, there is a growing interest in harnessing intelligent systems to analyze tongue data (Zhang et al., 2023; Gan et al., 2023). Although recent studies have made strides in image acquisition, segmentation, color calibration, and feature extraction, most such efforts remain confined to single-modality approaches relying exclusively on visual cues Zhang et al., 2021; Jiang et al., 2018). In real-world practice, however, TCM physicians not only inspect a patient’s tongue but also document critical observations—such as tongue color, coating, and shape—together with their professional judgment of pathological signs in textual form.

These textual descriptions often provide a more detailed and in-depth assessment of the appearance and potential pathological organs of the tongue. Therefore, the seamless integration of text and visual data not only brings tongue diagnosis closer to the clinical workflow, but also enables more accurate identification of pathological organs. However, little research has focused on systematically leveraging these complementary data sources in a unified framework.

In response to this need, this paper proposes a Cross-Modal Pathological Organ Diagnosis (CMPOD) model that unifies tongue images and textual information through a refined cross-attention mechanism based on the Transformer architecture. By fusing textual and visual features more precisely, the model seeks to achieve higher accuracy and robustness in identifying diseased organs. Departing from prior research, which typically processes images and text separately, our approach systematically incorporates textual notes from clinical practice, thus preserving both local and global pathological indicators. Specifically, we utilize Vision Transformer to capture image features and text descriptions are encoded using a transformer based bidirectional encoder, then fuse these representations using an enhanced cross-attention mechanism that avoids the shortcomings of simplistic weighting or direct concatenation.

Guided by established TCM principles mapping the abnormal lesions of the tongue to the corresponding internal organs, we further formulate the recognition task as a single-label classification problem covering heart–lung, liver–gallbladder, spleen–stomach, and kidney pathologies, closely mirroring actual diagnostic workflows. We conducted extensive comparative and ablation experiments on our self-constructed dataset. The results demonstrate that the proposed model effectively integrates image and text features, achieves strong performance in diagnosing pathological organs, and significantly outperforms single-modality methods.

This paper proposed a novel method (image-text fusion) for identifying visceral lesions through tongue diagnosis. By leveraging multimodal fusion, our approach not only enhances the objectivity and accuracy of TCM diagnostics but also serves as a bridge between traditional medical practices and state-of-the-art technologies. Moreover, it lays a foundation for developing intelligent diagnostic systems in personalized medicine, exploring for more accurate, efficient and accessible healthcare solutions. In addition, this study has important practical significance for pathological inference in clinical practice. By systematically analyzing the correlation between tongue features and visceral conditions, our model provides a more consistent and repeatable diagnostic tool that reduces reliance on subjective expert experience. This can enhance early disease detection and help practitioners make more informed decisions. By illustrating the potential of multimodal learning in medical applications, this work also advances artificial intelligence methodologies, offering novel insights into the integration of heterogeneous data for complex decision-making tasks.

## 2 Related work

### 2.1 Tongue diagnosis and machine learning

Tongue diagnosis has been a fundamental diagnostic method in TCM for thousands of years, playing an indispensable role in assessing patient health. According to TCM theory, tongue images change rapidly and significantly during the progression of disease (Rogers and Bruce, 2004), making the observation of tongue features a valuable non-invasive method for evaluating a patient’s physiological and pathological state. By examining characteristics such as tongue coating, texture, and color, practitioners can diagnose conditions related to internal organs and track the severity or progression of diseases (Li et al., 2020).

Clinical studies in TCM have established a systematic correlation between tongue regions and internal organs. For example, cardiopulmonary conditions are reflected at the tongue tip, spleen and stomach issues in the central area, kidney-related diseases at the root, and liver problems along the edges (Zhen, 2016). In the clinical study of tongue diagnosis, Li et al. (2014) randomly selected 50 confirmed cases of primary liver cancer, peptic ulcer, pulmonary/pleural tuberculosis, coronary heart disease, chronic glomerulonephritis and other diseases, observed the main manifestations of their tongue image changes, and found that the tongue image characteristics had a significant corresponding relationship with specific diseases. For example, 96% of patients with primary liver cancer had red and red tongue, 64% of patients with coronary heart disease had mushroom papillae hyperplasia in the anterior heart area of the tongue, and 82% of patients with chronic glomerulonephritis had gray or grayish-yellow thick fur at the base of the tongue (corresponding to the kidney area). These results clearly show that the pathological features of different diseases are significantly reflected in the tongue image, and the distribution law is highly consistent with the TCM tongue differentiation theory. These findings, viewed from the perspective of integrating TCM and Western medicine, further validate the connection between tongue features and internal organ pathology.

In previous research on tongue images, many efforts have targeted image collection (Lu et al., 2017), segmentation (Zhou et al., 2019), and color calibration (Wang and Zhang, 2013), enabling high-quality data for diagnostic applications. Beyond image preprocessing, various studies have addressed constitution identification, syndrome differentiation, and disease recognition. For example, Kanawong et al. (2012) verified the link between tongue color and syndromes using SVM, MLP, and RF, while Shi et al. (2021) integrated tongue and pulse data with four classifiers (random forests, logistic regression, SVM, and neural networks) to distinguish Qi deficiency from Yin deficiency. Ma et al. (2019) employed ResNet50 and VGG-16 for nine-constitution classification under varying difficulties, whereas Wen et al. (2024) embedded tensor reshaping and wavelet attention in ResNet18 to improve constitution recognition and attribute prediction. Zhang and Zhang (2014) used geometric features and sparse representation to separate healthy from diseased conditions, and Mansour et al. (2021) adopted ResNet50 to detect 12 diseases including nephritis, coronary heart disease, and verrucous gastritis. Although these works demonstrate the feasibility of automated tongue image analysis, most rely solely on image data, leaving textual descriptions largely unexploited.

However, current research has largely overlooked the textual descriptions of tongue images recorded by clinicians. Given the importance of textual records in clinical diagnosis, there is an urgent need to integrate textual information with tongue image features for more accurate diagnosis. In this paper, we propose using the ViT and Bidirectional Encoder Representations from Transformers (BERT) based on the Transformer, extract textual and image features, as well as employ an improved cross-attention mechanism to fuse them for enhanced diagnostic performance.

### 2.2 ViT, BERT, and cross-attention in transformer

Since the introduction of Transformer architecture (Vaswani et al., 2017), it has led to a series of landmark advances in various research fields, including natural language processing and computer vision. Models designed based on this architecture not only consistently outperform traditional methods in text understanding and generation tasks, but have also demonstrated superior abilities in image recognition and other visual tasks.

As typical applications of Transformer, ViT (Dosovitskiy, 2020), BERT (Devlin et al., 2019) and cross-attention mechanisms (Chen C.-F. R. et al., 2021) offer unique advantages in computer vision, natural language processing and multimodal tasks respectively. ViT is a model designed for computer vision tasks that captures long-range dependencies between pixels in an image, offering improved capabilities in complex image understanding. BERT is a model based on the encoder portion of the Transformer, used for natural language processing. By introducing bidirectional encoding, BERT excels in maintaining contextual coherence when processing both preceding and following text. The Cross-attention mechanism is a key attention mechanism in the Transformer decoder. Unlike traditional attention mechanisms, Cross-attention not only focuses on the current input of the target sequence but also attends to relevant information from the source sequence, enhancing the model’s ability to handle complex tasks.

Given Transformer’s advantages in capturing long-range dependencies and its attention mechanisms, several recent studies have applied Transformer models to medical diagnostic tasks. Particularly in the field of medical image recognition, significant progress has been made. For example, G. Van Tulder et al. used Transformer models to differentiate between COVID-19 and other forms of pneumonia in CT or X-ray images (Van Tulder et al., 2021), while Haoyuan Chen et al. combined Transformer models with CNNs for gastric tissue pathology image diagnosis (Chen et al., 2022). In tongue diagnosis, there are also studies exploring the application of Transformer models. Xinshen Zhao et al. combined ResNet with the Swin-Transformer module to classify tongue features such as tooth marks. Their model achieved high classification accuracy rates of 98.32% for mild, 97.92% for severe, and 98.90% for no tooth marks (Zhao et al., 2023). Furthermore, Baochen Fu et al. compared Transformer-based vision models with ResNet in diagnosing gastrointestinal diseases using tongue images, finding that the Transformer model achieved the highest recognition accuracy (Fu et al., 2024).

The core idea behind the Transformer is to determine relationships among sequence elements using a self-attention mechanism, which captures global information by examining the correlations between all elements. This capability endows Transformer-based models with enhanced reasoning and pathological diagnosis abilities compared to conventional approaches. Additionally, these models generate isomorphic feature representations—outputting sequence vectors with unified dimensions regardless of whether the input is an image or text—which naturally facilitates cross-modal fusion. This unified representation simplifies the application of cross-modal attention mechanisms without requiring additional transformations. In contrast, traditional methods often contend with heterogeneous feature spaces (e.g., spatial-structural features versus sequential features), necessitating complex alignment strategies during multimodal integration.

### 2.3 Multimodal fusion

Modality refers to the form or type of information or data, such as text, images, audio, and video, each of which has unique attributes and modes of expression. Multimodal Fusion involves integrating data from multiple modalities to achieve information complementarity and synergy across modalities, thereby enhancing the comprehensive utilization of data. In machine learning, multimodal fusion combines various data sources to extract richer features, improve reasoning capabilities, and enhance model performance.

In the medical field, multimodal fusion has also played a significant role. For example, F. Ali et al. combined sensor waveform data with electronic medical record text data, extracted features from both modalities, and employed information gain techniques for feature selection. They ultimately used an ensemble deep learning model to successfully diagnose heart disease (Ali et al., 2020). Additionally, M. Liu et al. integrated demographic information (text data) with MRI image data for Alzheimer’s Disease classification and clinical score regression, achieving remarkable results (Liu et al., 2018).

Multimodal fusion is generally categorized into feature-level fusion and decision-level fusion. Feature-level fusion, also known as early fusion, integrates data from different modalities before analysis to capture fine-grained semantic relationships between modalities. Decision-level fusion, also known as late fusion, entails independently training each modality and subsequently combining their prediction results during the decision-making stage.

In the multimodal fusion task of tongue image and textual description, specific regions in the images may have direct semantic connections with certain keywords in the text. In this paper, we adopt a feature-level fusion approach designed to capture these implicit cross-modal relationships and improve classification accuracy. By modeling cross-modal attention between image and text features within the feature space, our model effectively extracts both independent features and their interactions, allowing the classifier to leverage the complementary nature of the visual and textual information.

## 3 Materials and methods

### 3.1 Construction of dataset

#### 3.1.1 Data source and ethical considerations

**FIGURE 1:**
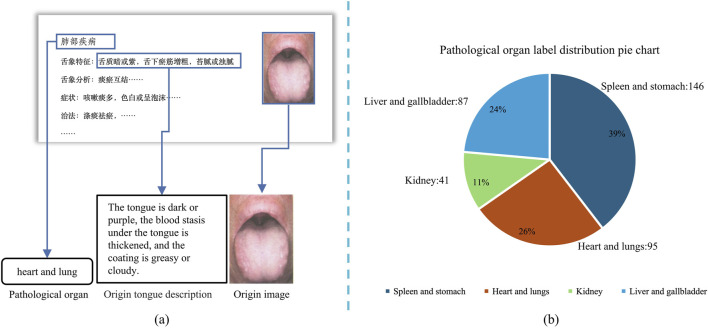
Data acquisition procedure and pathological organ label processing distribution pie chart, **(a)** shows the acquisition process of tongue image and corresponding text description, **(b)** shows the distribution of each label in the dataset.

As mentioned earlier, no research has specifically addressed the analysis of tongue images alongside their descriptive text, nor is there a publicly available dataset that meets the objectives of this study. Therefore, we compiled tongue-related images and descriptive text from publicly available literature (Zhang, 2017; Lai and Lai, 2017; Jia and Gu, 2008; Song and Song, 2022; Zhang, 2021; Chen et al., 2021b; Xu, 2017; Luo, 2018). The tongue images and their corresponding textual descriptions, as provided in the literature, are shown in Figure 1a.

It should be stated that these data are all derived from publicly available literature. According to relevant laws and academic norms, the reuse of such data for research purposes is appropriate. All sources have been duly acknowledged, and this study strictly adheres to the ethical principles established by the Declaration of Helsinki and the Declaration of Taipei in the process of data collection and usage.

#### 3.1.2 Composition and preprocessing of data

For the image data, we used high-resolution scanning equipment to digitize the original tongue images, ensuring high quality and resolution. Subsequently, the textual descriptions corresponding to each image were manually entered into the system to complete the pairing of images and text, thereby constructing the final sample dataset. To ensure data quality, we analyzed the resolution and color information of all images, confirming they met high-definition standards. Additionally, the textual data were thoroughly checked and reviewed to eliminate any missing or anomalous entries. Based on traditional theories regarding the correspondence between tongue features and internal organs, we categorized all samples into four groups: “Liver and Gallbladder,” “Spleen and Stomach,” “Heart and Lungs,” and “Kidneys.” Using the annotations provided in the original literature, we ensured the accuracy of this categorization process. The final dataset contains 95 Heart and Lungs samples, 89 Liver and Gallbladder samples, 44 Kidney samples, and 149 Spleen and Stomach samples, providing a reliable foundation for subsequent research. The label distribution of our dataset is shown in Figure 1b.

**FIGURE 2:**
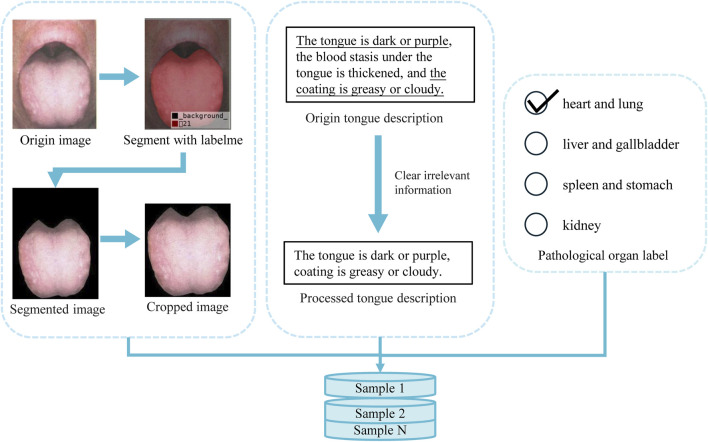
Pre-processing of original tongue image and the corresponding pathological organ label.

To prepare effective data samples for model training, we first extract the relevant regions from the raw data obtained through image scanning and text input. Consequently, as illustrated in Figure 2, proper preprocessing of the raw data is required.

In the collected tongue images, the meaningful tongue region occupies only a small portion, while areas outside the tongue will interfere with diagnosis. To address this, we used the LabelMe annotation tool to mark the effective tongue region. After annotation, the rest of the image was filled with a black background. We then cropped each image so that the tongue region would occupy the majority of the frame, leaving only minimal black background. Furthermore, to enhance data stability and reduce computational overhead, we normalized all images by scaling pixel values to the [0, 1] range. This step ensures consistency across samples and contributes to more efficient model training.

Each tongue sample is accompanied by a corresponding textual description, typically in the format “tongue texture, tongue coating, and additional features.” However, due to the complexity of real-world clinical diagnoses, not all descriptions strictly follow this structure. To conform to the actual diagnostic process, we retained the original wording and judgment methods used by professional doctors, preserving the authentic clinical records. This approach maintains data integrity while providing a sound clinical basis for model development.

Through these preprocessing steps, we effectively remove redundant information and noise, reducing the impact of irrelevant features and thereby improving the accuracy and efficiency of model training. After thorough preprocessing, the dataset is not only more consistent and standardized but also provides high-quality training samples, laying a strong foundation for subsequent model training.

To expand the dataset, and improve the generalization ability of the model, During training, we apply a series of random data augmentation techniques. We perform random horizontal flipping with a probability of 50%, rotation within ± 15°, and we randomly shuffle the above-processed dataset and divide it into 80% training set and 20% test set.

### 3.2 Evaluation metrics

In this paper, we use the Accuracy (ACC) (Equation 1), Precision (Equation 2), Recall (Equation 3), F1 (Equation 4) and ACC@O (Equation 5) as evaluation metrics. Among these equations, TP is the number of correctly assigned positive samples, FP is the number of incorrectly assigned positive samples, TN is the number of correctly assigned negative asmples,FN is the number of incorrectly assigned negative samples.

$$ACC=\frac{TP+TN}{TP+TN+FP+FN}$$ (1)

$$Precision=\frac{TP}{TP+FP}$$ (2)

$$Recall=\frac{TP}{TP+FN}$$ (3)

$$F1=\frac{2\times Precision\times Recall}{Precision+Recall}$$ (4)

ACC is the most intuitive evaluation metric, defined as the proportion of correctly predicted samples out of the total number of samples. Precision is defined as the proportion of true positive predictions among all positive predictions. It measures the model’s ability to avoid labeling negative samples as positive, thus reflecting the accuracy of the positive predictions. Recall is defined as the proportion of true positive predictions out of all actual positive samples. It evaluates the model’s ability to identify all relevant instances. F1 is the harmonic mean of precision and recall, with a higher indicating better model performance.

To further assess the model’s performance on different tongue image partitions, we introduce a custom evaluation metric called “ACC@O.” This metric is specifically designed to quantify the classification accuracy for a particular pathological organ and is defined as follows:

$$ACC@O=\frac{\sum_{i=1}^{No}I\left(y_{i}={\hat{y}}_{i}\right)}{N_{o}}$$ (5)

Here, O represents one of the following categories: “LG” (referring to the visceral organs Liver and Gallbladder), “SS” (Spleen and Stomach), “HL” (Heart and Lung), or “K” (Kidney). In this equation, $N_{O}$ denotes the total number of samples for organ O in the test set, $y_{i}$ is the true label of sample i , and ${\hat{y}}_{i}$ is the predicted label for that sample. The indicator function $I\left(y_{i}={\hat{y}}_{i}\right)$ equals 1 when the true label matches the predicted label and 0 otherwise. Thus, ACC@O represents the model’s classification accuracy for organ O , with a value closer to 1 indicating a stronger classification ability for that specific organ.

### 3.3 Cross-modal pathological organ diagnostic model

#### 3.3.1 Construction of model

**FIGURE 3:**
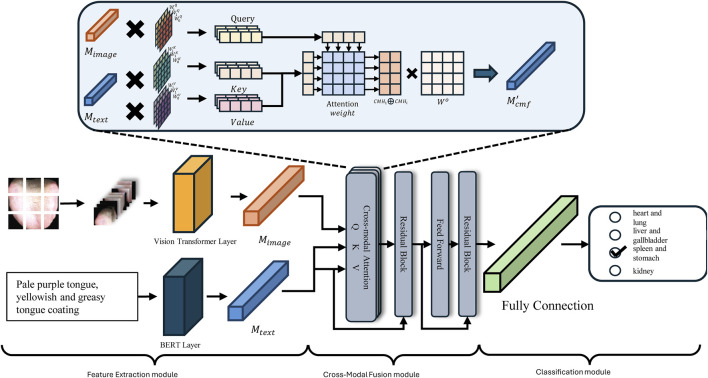
Model structure.

As discussed above, this study integrates ViT and BERT methodologies to develop a Cross-Modal Pathological Organ Diagnostic Model (CMPOD) in response to the current research landscape and the relevant technological advancements in tongue diagnosis. Figure 3 presents the framework of the proposed model. To facilitate the effective fusion of tongue images and textual descriptions, we have structured our approach into three primary modules: the Feature Extraction Module, the Cross-Modal Fusion Module, and the Classification Module. The following sections will detail the design principles, implementation strategies, and the respective roles of each module within the overall model architecture.

#### 3.3.2 Feature extraction module

In the feature extraction module, we need to encode the original images and texts to facilitate feature extraction. For the tongue image description, we utilize the BERT model for feature extractionas it excels at capturing bidirectional dependencies within sequences. Given the original text input $T_{\text{input}}=\left\{x_{1},x_{2},\ldots ,x_{t}\right\}$ , the BERT model generates text embeddings $H_{t}\in R^{t\times d}$ , where d is the output dimension of the BERT hidden layer, and t is the length of the input text sequence. The feature extraction process for the text input is represented by Equation 6.

$$M_{\text{text}}=BERT\left(T_{\mathrm{input}}\right)$$ (6)

For image input, we employ the ViT for feature extraction. Specifically, the input RGB image $I_{\text{input}}\in R^{H\times W\times C}$ is first divided into N image blocks (patches), each of size p×p . Each image block $I_{\text{patch},i}\in R^{p\times p\times C}$ is then mapped to an embedded vector $z_{i}\in R^{d}$ through a linear projection, where d is the embedding dimension. These image-embedding vectors undergo a multi-head self-attention process similar to that in Transformers to extract feature vectors. The feature extraction process for the image input is represented by Equation 7.

$$M_{\text{image}}=VisionTransformer\left(I_{\mathrm{input}}\right)$$ (7)

#### 3.3.3 Cross-modal fusion module

After extracting features from the input text and image, the Cross Modal Fusion (CMF) module performs the fusion of image and text features. At its core lies the cross-attention mechanism, which is an adaptation of the self-attention mechanism. In self-attention, the input features are projected into Query (Q), Key (K), and Value (V) matrices, and the dot product between Q and K is computed to obtain attention weights. In the cross-modal fusion module, we replace Q with the image features $M_{\text{image}}$ extracted from the image input, while K and V are represented by the text features $M_{\text{text}}$ . The specific steps are as follows:

I. Linear Transformation: After feature extraction, the image feature $M_{\text{image}}$ and the text feature $M_{\text{text}}$ are linearly transformed using learnable weight matrices $W_{Q}$ into Query $Q_{\text{image}}$ , Key $K_{\text{text}}$ , and Value $V_{\text{text}}$ respectively. The transformation process can be shown in Equation 8.

$$\left\{\begin{array}{c} Q_{\text{image}}=M_{\text{image}}W_{Q}\;\\ K_{\text{text}}=M_{\text{text}}W_{K}\;\\ V_{\text{text}}=M_{\text{text}}W_{V}\; \end{array}\right.$$ (8)

II. Multi-Head Attention: To capture multiple feature representations and accelerate parallel training, the module employs multi-head attention with each head having a dimension of $d_{h}=\frac{d}{h}$ , where h is the number of attention heads. This allows the model to obtain cross-modal attention $CMh_{i}$ for each head i , as defined in Equation 9.

$$CMh_{i}\left(M_{\text{text}},M_{\text{image}}\right)=Softmax\left(\frac{Q_{\text{image}}K_{\text{text}}^{⊤}}{\sqrt{d_{h}}}\right)V_{\text{text}}$$ (9)

III. Concatenation and Projection: As shown in Equation 10, the ouputs of all heads are concatenated and projected back into the original representation space to obtain the cross-modal attention CMA :

$$CMA\left(M_{\text{text}},M_{\text{image}}\right)=Concat\left[CMh_{1},CMh_{2},\ldots ,CMh_{h}\right]W^{O}$$ (10)

where $W^{O}\in R^{\left(h\cdot d_{h}\right)\times d}$ is a learnable projection matrix. This setup enables the image representation as a query to extract relevant text information from Key and Value, facilitating efficient alignment and fusion of multimodal features.

IV. Residual Connection and Normalization: Following the self-attention mechanism, to prevent the loss of original single-modal information during feature extraction, residual connections are employed. Specifically, the features from the previous layer are added to the extracted features, and the feature distribution is stabilized using a normalization layer LayerNorm(⋅) , the process feature $M_{cmf}^{\prime }$ is shown in Equation 11:

$$M_{cmf}^{\prime }=LayerNorm\left(M_{\text{image}}+CMA\left(M_{\text{text}},M_{\text{image}}\right)\right)$$ (11)

V. Feedforward Network and Final Representation: Finally, the output $M_{\text{cmf}}$ of the cross-modal representation is obtained by passing $M_{cmf}^{\prime }$ through a feedforward network and another residual connection followed by LayerNorm, the final output of Cross-modal fusion module M_cmf_ is shown in Equation 12.

$$M_{cmf}=LayerNorm\left(M_{cmf}^{\prime }+FFN\left(M_{cmf}^{\prime }\right)\right)$$ (12)

#### 3.3.4 Classification Module

The Classification Module generates the final prediction of the Pathological Organ based on the fused cross-modal representation $M_{\text{cmf}}$ . As shown in Equation 13. First, max pooling extracts the most salient features, producing $M_{\text{pooled}}$ . This pooled representation is then passed through a fully connected layer, which maps it to the class space via linear transformation. Finally, a Softmax activation function converts the logits into a probability distribution over K classes.

$${\hat{y}}_{k}=Softmax\left(FC\left(MaxPooling\left(M_{\text{cmf}}\right)\right),\;\forall k\in \left\{1,2,\ldots ,K\right\}\right.$$ (13)

During training, the model optimizes the classification performance using the Cross-Entropy loss (CE loss) for multi-class classification tasks. The CE loss is defined as Equation 14:

$$L=-\frac{1}{N}\overset{N}{\underset{i=1}{\sum }}\overset{K}{\underset{k=1}{\sum }}y_{ik}\log\left({\hat{y}}_{ik}\right)$$ (14)

where the N is the number of samples, $y_{ik}$ is the ground truth label for the k -th class of the i -th sample, and ${\hat{y}}_{ik}$ is the predicted probability.

The Classification Module effectively translates the rich, fused cross-modal features into actionable predictions. By leveraging max pooling, a fully connected layer, and the Softmax activation function, the module ensures that the model can accurately identify the presence of various Pathological Organs based on the combined image and text information.

## 4 Experiments and results analysis

This section presents experiments conducted to evaluate the CMPOD, aim to verify the effectiveness of the proposed model. The main components of this section include: Training Parameters; Comparative Experiments; Ablation Study.

### 4.1 Training parameter

In this paper, all experiments were conducted on a single NVIDIA GeForce RTX 3090 GPU (24 GB VRAM) using Python 3.8.10, PyTorch 2.2.1, and CUDA 12.1. We trained for 300 epochs with the AdamW optimizer (weight decay of 1e-2), starting at a 3e-4 learning rate reduced to 1e-6 via cosine annealing, and used a batch size of 64. During the training phase, we employ transfer learning strategies to reduce the dependency on extensive training data. Specifically, both the ViT and ResNet models, along with their variants, are initialized with pre-trained weights. The ViT models and their variants utilize weights pre-trained on the ImageNet-21k dataset (Dosovitskiy, 2020), while the ResNet models and their variants leverage weights pre-trained on the ImageNet-1k dataset (He et al., 2016).

### 4.2 Ablation experiments

To verify the effectiveness of our multimodal fusion approach, we conducted ablation experiments by comparing the full multimodal model with single-modal variants. In these experiments, we evaluated models where only one modality—the tongue image or the text description—was used for pathological organ diagnosis. For the single-modal settings, we extracted the CLS token from the final layer of either the VIT or BERT backbone, and fed it directly into the classifier. Table 1 summarizes the performance of the full multimodal model alongside the image-only and text-only variants.

**TABLE 1:** Results of ablation study, bold numbers indicate the best performance for each metric.

Model	ACC	Precision	Recall	F1
Full	**62.90** ±2.10	**54.87** ±3.38	**55.03** ±2.23	**54.17** ±2.33
-image only	43.42 ±1.98	31.88 ±2.37	35.89 ±2.18	33.60 ±2.53
-text only	56.58 ±1.53	42.21 ±2.98	48.27 ±1.88	42.54 ±2.72

The results demonstrate that the classification performance of both single-modal models is considerably inferior to that of the full multimodal model. More specifically: (1) When only tongue images are used, the model’s performance drops markedly (ACC of 43.42%), indicating that image features alone are insufficient to fully capture the complex characteristics of the pathological areas. (2) When only tongue text descriptions are used, the performance also declines significantly (with an accuracy of only 56.58%), indicating that relying solely on textual information—given the brevity and similarity of the descriptions—makes it difficult to distinguish subtle pathological differences.

The ablation study results reveal that the substantial gap between the single-modal and multimodal outcomes highlights the strength of our CMPOD model’s design, demonstrating that our model can effectively extract and integrate features essential for accurate pathological organ diagnosis. Furthermore, these results underscore the importance of effectively fusing tongue images and text descriptions in multimodal tasks, providing empirical evidence that combining multiple data sources not only enriches the feature space but also enhances the overall generalization ability of the model.

### 4.3 Comparative experiments and their results

To our knowledge, there have been no prior studies on multimodal pathological organ diagnosis utilizing both text and image data. To explore the impact of different image feature extraction methods, we replaced the image feature extraction component of our model with several traditional and advanced architectures. Specifically, we utilized various variants of the ViT model, including ViT-Small and ViT-Base, as well as multiple ResNet variants, such as ResNet-18, ResNet-34 and ResNet-50. Additionally, we incorporated VGG model variants, including VGG-16 and VGG-19. These models were applied to our established dataset for pathological organ diagnosis, providing a comprehensive evaluation of their performance. All experimental results are averaged over five runs.

**FIGURE 4:**
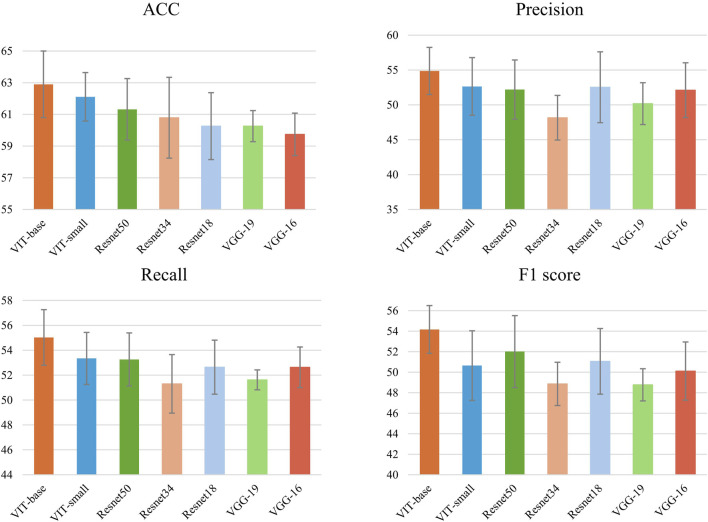
Accuracy, recall,precision and F1 of methods with 5 runs for pathological organ recognition.

In Table 2; Figure 4, the comparative results of seven models on four key metrics are presented. Overall, the CMPOD-ViT-base model exhibits the best performance, achieving 62.90% (ACC), 54.87% (Precision), 55.03% (Recall), and 54.17% (F1-score), while the other models perform slightly worse on all metrics. Meanwhile, CMPOD-small follows closely with an accuracy of 62.11%, its Precision, Recall, and F1-score are all lower than those of ViT-base, suggesting that reducing the model size may compromise stability or generalization ability. Within the ResNet series, CMPOD-ResNet50 performs the best (61.32% ACC, 52.20% Precision, 53.26% Recall, and 52.01% F1-score), yet it still lags compared to the ViT series. Furthermore, CMPOD-VGG-16 and CMPOD-VGG-19 performed the worst, with VGG16’s accuracy falling below 60%.

**TABLE 2:** Comparative experimental results of four key metrics, bold numbers indicate the best performance for each metric.

Model	ACC	Precision	Recall	F1-score
CMPOD-ViT-base	**62.90** ±2.10	**54.87** ±3.38	**55.03** ±2.23	**54.17** ±2.33
CMPOD-ViT-small	62.11 ±1.53	52.65 ±4.13	53.34 ±2.09	50.64 ±3.40
CMPOD-ResNet50	61.32 ±1.94	52.20 ±4.24	53.26 ±2.13	52.01 ±3.51
CMPOD-ResNet34	60.79 ±2.55	48.16 ±3.20	51.30 ±2.35	48.86 ±2.11
CMPOD-ResNet18	60.26 ±2.11	52.53 ±5.08	52.64 ±2.17	51.06 ±3.20
CMPOD-VGG-19	60.26 ±0.98	50.18 ±3.00	51.62 ±0.80	48.77 ±1.57
CMPOD-VGG-16	59.74 ±1.34	52.11 ±3.93	52.63 ±1.63	50.11 ±2.84

In Table 3, which shows the accuracies of seven models on different pathological organs using our custom ACC@O metrics, the CMPOD-ViT-base model exhibits the best performance with classification accuracies of 60.00% on the heart/lung (ACC@HL) and 30.00% on the kidney (ACC@K). Comparatively, the CMPOD-ViT-small model achieves the highest accuracy on the liver/gallbladder (ACC@LG) at 74.12%, while CMPOD-ResNet50 performs well on the spleen/stomach (ACC@SS) with 83.57%. Overall, the ViT-base model performs particularly well, with its ACC@HL and ACC@K metrics significantly outperforming those of the other models. Although its ACC@LG and ACC@SS values are not the highest, they remain quite promising. However, all models show relatively low classification accuracy on the kidney (ACC@K).

**TABLE 3:** ACC@O of proposed method and several compare method, bold numbers indicate the best performance for each metric.

Model	ACC@LG	ACC@SS	ACC@HL	ACC@K
CMPOD-ViT-base	64.71	81.43	**60.00**	**30.00**
CMPOD-ViT-small	**74.12**	82.86	52.38	24.00
CMPOD-ResNet50	61.18	**83.57**	54.29	14.00
CMPOD-ResNet34	63.53	82.14	51.43	6.00
CMPOD-ResNet18	70.59	80.71	51.43	8.00
CMPOD-VGG-19	71.77	82.14	48.57	4.00
CMPOD-VGG-16	77.65	77.14	45.71	10.00

**FIGURE 5:**
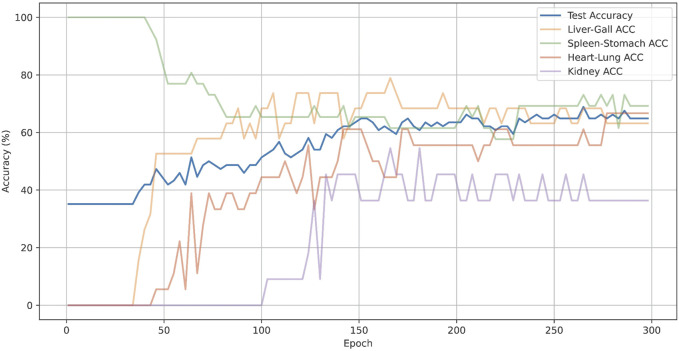
Accuracy and the changes in ACC@O over epochs.

Also, we have plotted the overall accuracy and the changes in ACC@O over epochs of CMPOD-VIT-base, as shown in Figure 5. In the Figure 5, the blue lines represent the overall accuracy, while other color lines represent the ACC@O of the model. The model’s overall accuracy peaked at around 200 epochs, although the accuracy for each part exhibited significant dynamic fluctuations. For instance, the accuracy for the spleen and stomach parts was highest during the initial stages and subsequently dropped to 74% as the model gradually balanced the feature learning across different parts. In contrast, the accuracy for the kidney began to increase only in the later stages of training, but it ultimately remained low.

**FIGURE 6:**
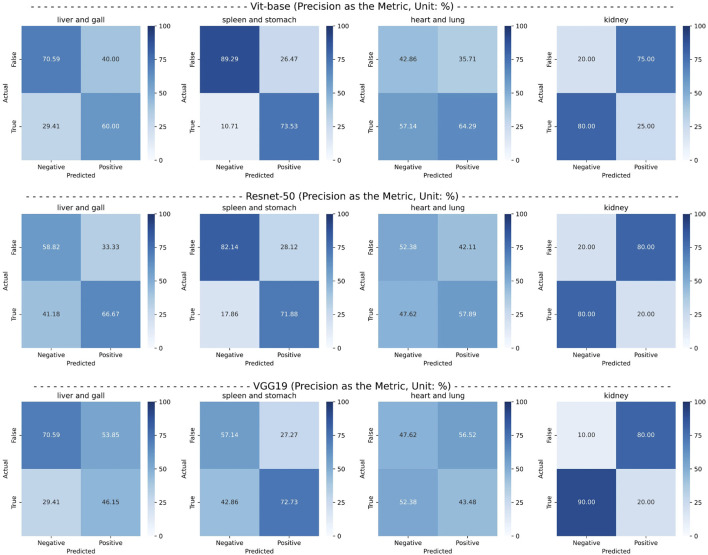
Confusion matrix of the best-performing models in each series, using precision as an metric.

Finally, from the ViT series, ResNet series, and VGG series, we selected the top-performing models—ViT-base, ResNet50, and VGG19—and conducted confusion matrix experiments for each pathological organ. The experimental results are presented in Figure 6. It can be observed that the CMPOD model outperforms the other models in most categories.

Based on the results of comparative experiments, we can draw the following conclusions: (1) The CMPOD model demonstrates robust and balanced performance in most pathological organ classification tasks. In particular, it achieves high accuracies for the heart/lung (ACC@HL) and kidney (ACC@K), confirming the effectiveness and reliability of our approach. (2) Although ResNet and VGG models perform reasonably well in some organ categories, they generally fall short in multimodal pathological organ diagnosis, often performing significantly worse than the CMPOD model. (3) All models, including CMPOD, exhibit relatively low accuracy on the kidney (ACC@K). This may be due to either an insufficient number of kidney samples or the inherent difficulty of extracting kidney-related features from tongue images.

Overall, the results confirm the effectiveness and superiority of our CMPOD model in multimodal pathological organ diagnosis, while also highlighting current limitations that point to clear directions for future optimization and dataset expansion.

## 5 Conclusion and discussion

In this study, we address the challenge posed by traditional TCM tongue diagnosis, which heavily relies on subjective clinical experience and lacks standardized, objective evaluation methods. Recognizing that current research on intelligent tongue diagnosis has largely overlooked the importance of integrating tongue images with their corresponding textual descriptions, we developed a multimodal deep learning approach for pathological organ diagnosis. Our approach focuses on integrating tongue images and descriptive texts into a unified framework, termed the Cross-Modal Pathological Organ Diagnosis (CMPOD) model. To achieve this, we first employ a ViT for image feature extraction and BERT for textual feature extraction. Next, a cross-attention mechanism is utilized to effectively fuse these multimodal features, and finally, the fused representations are passed to a fully connected classifier for end-to-end diagnosis. To evaluate the performance of our proposed model, we conducted extensive comparative experiments and ablation studies. In the comparative experiments, several baseline models—including various ViT, ResNet, and VGG variants—were tested on the same dataset. The results demonstrate that our model achieves higher classification accuracy in key categories and overall outperforms the baseline models. In the ablation studies, we compared single-modal approaches (using only tongue images or only text descriptions) against the full CMPOD model. The findings reveal that relying solely on tongue images or text descriptions results in a substantial performance drop, thereby underscoring the critical importance of multimodal feature fusion.

This study addresses a gap in previous research by focusing on the often-overlooked textual information in tongue image descriptions. We introduce a cross-modal attention mechanism that fuses textual and visual features, thereby enhancing diagnostic accuracy. This complementary diagnostic approach not only enriches the decision-making process but also better aligns with clinical practices by integrating multi-source evidence. In practical applications, our method can be incorporated into TCM clinical diagnostic systems, allowing clinicians to rapidly analyze tongue images from a large number of patients. This integration enhances diagnostic efficiency and alleviates clinicians’ workload. Furthermore, our work supports the digital and intelligent transformation of TCM, offering promising applications in telemedicine and mobile health monitoring systems. On the other hand, the adoption of AI-driven TCM diagnosis may raise ethical issues. The training process often involves sensitive medical data, making it crucial to implement measures such as data desensitization and encrypted storage to safeguard patient privacy. Additionally, the inherent “black box” nature of many AI models can obscure the decision-making process, potentially eroding clinical trust. To address these concerns, it is essential to incorporate interpretable algorithms that enhance transparency and accountability in the diagnostic process.

Although this study is the first to propose a diagnostic model that integrates tongue images and descriptive texts through multimodal fusion—and the experimental results are promising—our approach still has some limitations that merit further investigation. (1) While the CMPOD model demonstrates robust performance across most pathological organs, its classification accuracy for the kidney remains relatively low. Notably, a previous study on disease location classification using tongue images [e.g., Hu et al. (2021)] reported a similar trend, where kidney sites yielded lower accuracy compared to other sites. This limitation may be attributed to two factors: the limited number of kidney samples in the dataset and the inherent difficulty of capturing subtle kidney-related features from tongue images. In particular, since the kidney’s reaction area is located at the root of the tongue, acquiring high-quality images of this region is especially challenging, further complicating the extraction of relevant features. (2) All data used were sourced from existing literature. Although we have proposed a novel model based on these data, the diversity of real-world clinical data may affect the model’s generalizability. (3) Although our model outperforms other approaches overall, performance discrepancies persist across different organ categories. This suggests that the model’s generalization ability requires further optimization and fine-tuning to fully leverage the complementary information from multiple modalities. Future work will focus on several key areas: (1) Expanding and refining the dataset, particularly by increasing the number of kidney samples, is crucial to improving the recognition of challenging organs. (2) Further refining the model architecture and exploring advanced fusion techniques may enhance both performance and stability. (3) Investigating alternative optimization strategies and regularization techniques is essential for reducing overfitting and improving the model’s generalizability across diverse pathological cases. (4) Gathering data from real-world clinical diagnoses to further refine and optimize the model, thereby enhancing its generalizability.

## Data Availability

The original contributions presented in the study are included in the article/supplementary material, further inquiries can be directed to the corresponding author.
